# Yellapragada Subbarow: A Pioneer in Biomedical Research and the Unsung Hero of Modern Medicine

**DOI:** 10.7759/cureus.67442

**Published:** 2024-08-21

**Authors:** Mayank Sharma, Sonali G Choudhari, Abhishek Ingole

**Affiliations:** 1 Community Medicine, Jawaharlal Nehru Medical College, Datta Meghe Institute of Higher Education and Research, Wardha, IND

**Keywords:** historical vignette, tetracycline antibiotics, chemotherapy, folic acid, atp discovery, biomedical research, yellapragada subbarow

## Abstract

Yellapragada Subbarow, an often overlooked yet monumental figure in biomedical research, made groundbreaking contributions that have profoundly shaped modern medicine. Born in 1895 in Andhra Pradesh, India, Subbarow's journey from humble beginnings to a pioneering biochemist is a testament to his relentless determination and intellectual prowess. His discoveries, including the elucidation of adenosine triphosphate (ATP), the development of folic acid, and the introduction of methotrexate and tetracycline antibiotics, have had a lasting impact on various fields such as biochemistry, oncology, and infectious disease treatment. Despite his significant scientific achievements, Subbarow's name remains relatively obscure outside academic circles. This review highlights his pivotal contributions and explores the reasons behind his underrecognition. By examining his life's work, this article seeks to celebrate Subbarow's enduring legacy and advocate for greater recognition of his contributions to medical science. His story enriches our understanding of scientific progress and is an inspiring example of the profound impact of perseverance and innovation in advancing human health. Through this review, we hope to honor Subbarow's remarkable achievements and bring deserved attention to one of the unsung heroes of modern medicine.

## Introduction and background

**Figure 1:**
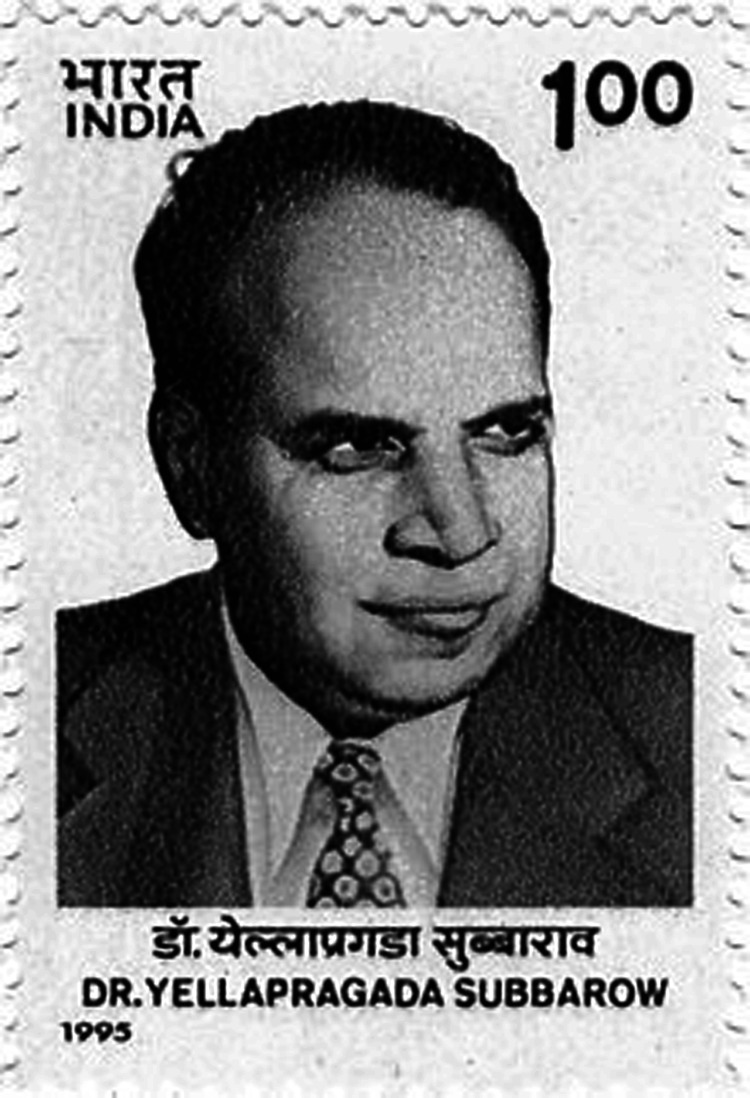
Yellapragada Subbarow Image Credit: This is an open-source image. (This work is in the public domain in India because its copyright term has expired.)

Yellapragada Subbarow remains a pivotal figure in the annals of biomedical research, yet his contributions are often overlooked in mainstream narratives of scientific history (Figure 1) [1]. Born in 1895 in the Indian state of Andhra Pradesh, Subbarow's early life was marked by a keen intellect and an insatiable curiosity for science. Despite facing numerous hardships, including financial constraints and the untimely loss of his father, Subbarow pursued his education with relentless determination [1]. His journey from a modest upbringing to becoming a pioneering biochemist and researcher exemplifies perseverance and dedication amidst formidable challenges [1].

Throughout his career, Subbarow made seminal contributions that significantly advanced our understanding of biochemistry and medicine. He is credited with the discovery of adenosine triphosphate (ATP), a molecule essential for cellular energy transfer, and played a crucial role in developing folic acid and methotrexate, a cornerstone of chemotherapy. Additionally, his work led to the discovery of tetracycline antibiotics, revolutionizing the treatment of bacterial infections. These breakthroughs laid the groundwork for crucial developments in chemotherapy, antibiotics, and the treatment of nutritional deficiencies, profoundly impacting global healthcare practices [2].

This article aims to illuminate the groundbreaking work of Yellapragada Subbarow and underscore his status as an unsung hero of modern medicine. Despite his profound influence on medical science, Subbarow's name remains relatively obscure outside academic circles. By highlighting his extraordinary achievements and examining the factors contributing to his underrecognition, this review seeks to bring deserved attention to his legacy. In exploring Subbarow's scientific journey, we aim to inspire a deeper appreciation for his contributions and advocate for broader recognition of his pivotal role in shaping contemporary biomedical research. His story enriches our understanding of scientific progress and serves as a testament to the enduring impact of dedication and innovation in advancing human health. Through this article, we hope to celebrate Subbarow's remarkable legacy and encourage the acknowledgment of other overlooked pioneers in the history of science and medicine.

## Review

Early life and education

Yellapragada Subbarow, born on January 12, 1895, in Bhimavaram, Andhra Pradesh, India, was a pioneering biochemist whose early life and education laid the foundation for his remarkable contributions to modern medicine [3]. He was born into a Telugu Brahmin family; his father, Jagannatham, worked as a revenue inspector, while his mother, Venkamma, was a traditional housewife [3]. The family faced financial difficulties, which led to a hand-to-mouth existence. Subbarow was one of eight siblings, and the tragic loss of two brothers to tropical diseases during his youth had a profound impact on him, instilling a desire to pursue a career in medical research [3]. Subbarow's academic journey was fraught with challenges. He struggled in school, flunking out of two high schools before finally passing his matriculation examination on his third attempt at Hindu High School in Madras. His interest in science, particularly mathematics and chemistry, blossomed during his intermediate studies at Madras Presidency College. After completing his studies, he enrolled at Madras Medical College in 1915, where he developed a keen interest in medical research, motivated by the tragic deaths of his brothers from tropical sprue [4].

In 1923, Subbarow moved to the United States to further his education and research opportunities. His initial medical degree from India was not recognized in the U.S., prompting him to seek additional qualifications [5]. Financial support from friends and his father-in-law enabled him to pursue this goal. Upon arriving in Boston in 1922, Subbarow faced numerous hardships, including working as a night porter at a hospital to support himself. Despite these challenges, he enrolled in the Harvard School of Tropical Medicine, where he continued his research in biochemistry. His collaboration with Cyrus Fiske led to the development of the Fiske-Subbarow method for estimating phosphorus in biological samples, a significant contribution that would have lasting implications in the field of biochemistry [5]. Subbarow's early experiences in the U.S. were marked by determination and resilience, setting the stage for his later groundbreaking medical discoveries. His journey from a small town in India to the forefront of biomedical research showcases not only his intellect and dedication but also his unwavering commitment to improving human health through science [6].

Major scientific contributions

Yellapragada Subbarow made several significant scientific contributions that have impacted biochemistry, medicine, and pharmacology. He is credited with discovering ATP as a vital energy carrier in cells [7]. ATP is often referred to as the "energy currency" of the cell, facilitating energy transfer necessary for various cellular processes. Subbarow's work demonstrated how ATP is generated and utilized in muscle contraction and other metabolic functions, highlighting its critical role in biochemistry and physiology. The identification of ATP fundamentally changed living organisms' understanding of energy metabolism. Subbarow's research established the relationship between ATP and phosphocreatine, underscoring the mechanisms by which cells store and utilize energy. This discovery has become a cornerstone in biochemistry, influencing countless studies and applications in cellular biology [4]. Subbarow also developed a method for synthesizing folic acid, which is essential for DNA synthesis and repair. His work was pivotal in isolating folic acid to treat nutritional deficiencies, particularly tropical sprue, which affected many individuals in his native India. Folic acid, also known as Vitamin B9, plays a significant role in preventing and treating anemia. Subbarow's contributions to the understanding of folic acid's importance have led to its widespread use in clinical settings to address various health issues, including anemia and other nutritional deficiencies [1].

Furthermore, Subbarow was instrumental in developing methotrexate, one of the first effective chemotherapy drugs. Working alongside Dr. Sidney Farber, he helped create this antifolate drug, which inhibits the metabolism of folic acid, thereby slowing the growth of cancer cells. Methotrexate has become a cornerstone in cancer treatment, used for various malignancies, including leukemia and lymphoma. Additionally, it is utilized in treating autoimmune diseases such as rheumatoid arthritis, showcasing its versatility and importance in modern medicine [8]. Subbarow also led the research at Lederle Laboratories, where his team discovered chlortetracycline, the first tetracycline antibiotic. This discovery was crucial in developing antibiotics that effectively combat bacterial infections. Tetracycline antibiotics have profoundly impacted treating a wide range of bacterial infections, making them essential tools in medical practice. Subbarow's contributions to antibiotic research have saved countless lives and continue influencing antibiotic therapy today [9]. Through these key discoveries, Yellapragada Subbarow significantly advanced the fields of biochemistry and medicine, leaving a legacy that continues to benefit humanity [9].

Challenges and obstacles

Yellapragada Subbarow's journey in the field of biomedical research was marked by numerous challenges and obstacles he had to navigate throughout his career. These struggles not only shaped his professional life but also had profound effects on his personal life [8]. One of the most significant challenges Subbarow faced was the lack of recognition for his groundbreaking scientific contributions. Despite his pivotal role in discovering folic acid and developing antimalarial drugs, he often found himself overshadowed by more prominent figures in the scientific community. This lack of acknowledgment was particularly frustrating, as many of his discoveries were later celebrated and credited to others. The competitive nature of scientific research, combined with the prevailing biases of the time, meant that Subbarow's work did not receive the acclaim it deserved during his lifetime [3].

Subbarow also encountered financial and institutional hurdles that complicated his research efforts. As a scientist in a developing country, he often struggled to secure funding for his projects. Limited access to advanced research facilities and resources made it difficult for him to conduct experiments at the level required to fully validate his theories. These financial constraints hindered his ability to collaborate with other researchers and institutions, which could have amplified the impact of his work [10]. The demands of his research took a toll on Subbarow's health and personal life. The intense focus required for his work often meant long hours in the lab and little time for relaxation or personal relationships. This dedication, while admirable, led to significant stress and health issues, which he had to manage while continuing to pursue his scientific goals. The sacrifices he made in his personal life are a testament to his commitment to advancing medical science [10].

Despite these numerous challenges, Subbarow's perseverance and determination were remarkable. He faced setbacks with resilience, continuing to push the boundaries of scientific knowledge even when recognition and support were lacking. His unwavering commitment to his research and belief in its potential impact on public health drove him to overcome obstacles that would have deterred many others. This tenacity contributed to his eventual success and was an inspiring example for future scientists [10]. Yellapragada Subbarow's life story is one of remarkable achievements against significant challenges. His professional struggles with recognition, institutional support, and personal sacrifices highlight the complexities many scientists face. Ultimately, Subbarow's legacy is a powerful reminder of the importance of perseverance in the pursuit of knowledge and the impact that one dedicated individual can have on the world of medicine [10].

Legacy and recognition

Yellapragada Subbarow's contributions to biomedical research have had a lasting impact on modern medicine, yet he remains a lesser-known figure than his contemporaries. Significant scientific advancements, posthumous recognition, and a unique position among medical pioneers characterize his legacy [1]. Subbarow's discoveries, particularly in biochemistry and pharmacology, have had profound implications for medical science. His work on ATP established it as a crucial energy molecule in cellular processes, fundamentally altering our understanding of metabolism and energy transfer in biological systems. Furthermore, his identification of folic acid's role in cell division and its application in cancer treatment has been pivotal in chemotherapy protocols, particularly in developing antifolate drugs like methotrexate. Subbarow's methodologies and findings continue to influence contemporary research, with his colorimetric determination of phosphorus remaining a foundational technique in biochemical analysis and the ongoing exploration of folic acid's health benefits underscoring his lasting impact on nutrition and medicine [11].

Despite facing significant challenges during his lifetime, Subbarow has received various posthumous honors. In 2003, the Government of India issued a commemorative stamp in his honor, and Vigyan Prasar published a biography detailing his life and contributions. His legacy is also celebrated through various articles and discussions advocating for greater recognition of his work. Institutions and awards have been established to honor Subbarow's contributions, such as a genus of fungi, *Subbaromyces*, named after him, reflecting his impact on biological sciences [12]. Subbarow's contributions place him among the ranks of significant medical pioneers; however, he has not received the same recognition as figures like Paul Ehrlich or Selman Waksman, who are celebrated for their discoveries in antibiotics. His work on antibiotics, particularly tetracycline, is crucial yet often overshadowed by his contemporaries who received Nobel Prizes for similar achievements. Several factors contribute to Subbarow's lesser-known status, including discrimination and lack of institutional support during his career, his reluctance to publish under the names of others, and the cultural and historical context of his work, combined with the overshadowing fame of his contemporaries [13].

## Conclusions

Yellapragada Subbarow's remarkable journey from a small village in India to the forefront of biomedical research epitomizes the profound impact of perseverance, intellect, and innovation. His groundbreaking discoveries, including ATP, folic acid, methotrexate, and tetracycline antibiotics, have left an indelible mark on modern medicine, revolutionizing the treatment of diseases and enhancing our understanding of cellular processes. Despite his significant contributions, Subbarow's legacy remains underappreciated, overshadowed by more prominently recognized figures in the field. This article has sought to illuminate his extraordinary achievements and highlight the importance of acknowledging the work of unsung heroes in science. By honoring Yellapragada Subbarow's legacy, we not only pay tribute to a pioneer who transformed medical science but also inspire future generations to recognize and celebrate the diverse array of contributors who shape our understanding of health and disease. Subbarow's life and work stand as a testament to the enduring power of scientific inquiry and the critical need for equitable recognition in the annals of scientific history.
